# Tenofovir diphosphate in dried blood spots and HIV-1 resistance in South Africa

**DOI:** 10.1186/s12981-023-00552-w

**Published:** 2023-09-14

**Authors:** Y. Singh, J. Castillo-Mancilla, R. Madimabe, L. Jennings, C. M. Ferraris, R. N. Robbins, P. L. Anderson, R. H. Remien, C. Orrell

**Affiliations:** 1 Desmond Tutu HIV Centre, Institute of Infectious Diseases and Molecular Medicine and Department of Medicine, University of Cape Town, Cape Town, South Africa; 2 University of Colorado, Aurora, CO USA; 3 New York State Psychiatric Institute, New York, USA; 4 Columbia University Irving Medical Center, New York, USA

**Keywords:** South Africa, HIV resistance, Adherence, Dried blood spots, Tenofovir diphosphate

## Abstract

**Background:**

Suboptimal antiretroviral (ART) adherence can lead to virologic failure with consequent HIV-1 resistance. Tenofovir diphosphate (TFV-DP) in dried blood spots (DBS) is a powerful biomarker of cumulative adherence, predictive of future viremia. It has been associated with resistance in Persons With HIV (PWH) in South Africa and the US. We explored the relationship of TFV-DP concentrations with antiretroviral drug resistance at the time of treatment failure in SA.

**Methods:**

Adult PWH from health clinics in Cape Town, South Africa on efavirenz-based first-line ART containing tenofovir disoproxil fumarate (TDF) with an undetectable (< 50 copies/mL) HIV-1 viral load (VL) were prospectively enrolled in an observational cohort for 12 months. Monthly study visits included blood collection for HIV-1 VL and DBS for TFV-DP. The first confirmed viral breakthrough (VB) > 400 copies/mL triggered HIV-1 genotyping at the subsequent visit. An electronic adherence (EA) device monitored ART adherence in real-time, estimated as a percent for the 30-days prior to VB. Wilcoxon rank sum test was used to compare median [IQR] TFV-DP by genotype outcome.

**Results:**

Of 250 individuals, (n = 195, 78% women), 21 experienced VB, with a median of 5 [4;7] months on study, and a median EA of 33.3 [13.3;53.3]%. Demographic characteristics between those with and without VB were similar. Median VL at VB was 4.0 [3.2;4.5] log copies/mL. TFV-DP concentrations trended down towards the VB visit. Median TFV-DP concentrations were significantly higher in those HIV-1 genotype did not amplify due to being virally suppressed at the subsequent visit (n = 10; 380 [227–661] fmol/punch, p = 0.035; EA 45 [24.9; 59.2]%); than in those who were successfully genotyped with evidence of drug resistance (n = 5, 241 [150–247] fmol/punch, EA 20 [6.7;36.7]%) and in individuals who did not have resistance (n = 3, 39.9 [16.6; 93.9] fmol/punch; EA 33.3 [16–38]%). Three genotype collections were not done. Only non-nucleoside reverse transcriptase inhibitor-associated mutations were identified on resistance testing. (K103N, E138K, Y118H).

**Conclusion:**

TFV-DP in DBS showed a step-wise inverse relationship with VB and drug resistance, with evidence of low cumulative ART adherence in PWH who developed antiretroviral resistance. Monitoring TFV-DP concentrations could be a valuable tool for predicting future VB and future resistance.

## Background

Maintaining viral suppression in PWH is the objective of ART [1, 2] and is crucial in preventing transmission of HIV-1 (Human Immunodeficiency Virus Type-1) [3] and adverse clinical and virologic outcomes [2, 4, 5]. Newer regimens are more pharmacologically forgiving, have fewer toxicities and may be co-formulated, all which support an individual’s ability to adhere to treatment, and remain virally suppressed [6, 7]. However, lifelong adherence is complex and subject to both individual and contextual factors, such as treatment fatigue, stigma and access to care [8]. In addition, periods of treatment interruption are associated with increased inflammatory biomarkers even in individuals who are suppressed, placing them at higher risk for long term chronic illnesses [9, 10].

Traditional adherence tools used in resource limited settings such as pharmacy refill data, pill counts and self-reported adherence vary in diagnostic accuracy [11]. A more reliable measure of pill-taking behaviour, shown to correlate with VL, is real-time electronic adherence monitoring [11–13]. Wisepill®, an example of an electronic pillbox, provides real-time tracking over cellular networks, with added text message reminders if a device-opening signal is not received [14, 15].

The quantification of TFV-DP concentrations in DBS samples as an adherence biomarker is gaining traction [1, 2, 6, 16]. TFV-DP is the anabolite of TFV which is phosphorylated in red blood cells [6] and has an intracellular half-life of 17 days [6, 16]. TFV-DP in DBS is informative of cumulative TDF dosing in the preceding 6–8 weeks [17]. Most first-line regimens in South Africa contain TDF, [18] hence TFV-DP assessment in DBS is an attractive method to accurately assess adherence in South African PWH.

Researchers in the US established that concentrations of TFV-DP in DBS strongly correlate with viral suppression (VL < 20 copies/mL) in PWH on a TDF-based regimen [1]. Expanding on this, Morrow et al. demonstrated how this biomarker can predict viral rebound (VL > 20 copies/mL) even in suppressed PWH at the time of the assessment [2]. The ADD-ART study (Use of ARV Drug Levels in DBS to Assess and Manage ART Adherence in South Africa) is the first to define TFV-DP concentrations predicting VB (VL > 400 copies/mL) in a South African population, establishing this threshold to be < 400 fmol/punch [6].

In individuals with suboptimal cumulative adherence, drug selection pressure drives the mutation of treatment resistant virus, which may be accelerated by the use of non-nucleoside reverse transcriptase inhibitors (NNRTIs) [19, 20]. A study conducted in South Africans on a NNRTI regimen with virologic failure described a step-wise decrease in TFV-DP concentrations both with and without evidence of drug resistance [21]. Those with drug resistance had mid-range TFV-DP concentrations, compared to individuals without resistance [21]. This further highlights the potential of TFV-DP in DBS for early detection of individuals at risk for developing drug resistance. In comparison with this research, the utilisation of Electronic Monitoring (EM) in our study provided data from two objective measures of adherence.

In South African clinical practice, VL measurement is the standard proxy for acceptable adherence, and this is only measured annually in already suppressed PWH [18]. Since VB can develop months after a decrease in ART adherence, a suppressed VL gives little insight into preceding and future adherence [4, 22, 23]. This underscores the need for more refined monitoring tools, giving a more nuanced picture than suppressed versus non-suppressed. EM and TFV-DP in DBS may enable providers to initiate early adherence support in those at risk of virologic failure [2, 6, 15]. Further investigation of the relationship between TFV-DP concentrations and VB is needed.

Extending on findings from ADD-ART [6], we present a descriptive analysis of the participants who met the endpoint for VB in the study, across categories of virologic failure. We explored adherence using an integrative approach of both EM and monthly TFV-DP sampling.

## Methods

### Aim, design and setting

This research examines a subpopulation from the previously published parent study [6]. The ADD-ART study was a prospective observational study conducted in Gugulethu, Cape Town. The main objective of ADD-ART was to determine the range of TFV-DP concentrations in DBS associated with viral suppression, and to compare the ability of DBS TFV-DP and electronic adherence (EA) to predict VB [6].

Between November 2016 and November 2018, the study enrolled 250 PWH from 4 public healthcare clinics. Eligible individuals had been receiving a TDF-based regimen for at least 4 months and for less than 24 months. For those who were treatment experienced (on ART for ≥ 12 months), evidence of historic adherence challenge was required (defined as: any previous VL > 400 copies/mL, or < 90% adherence on pharmacy refill data or pill count calculations in the past 12 months). Individuals had to be virally suppressed (VL < 50 copies/mL) a month prior to enrolment. All 250 participants were taking a single tablet regimen containing tenofovir, emtricitabine and efavirenz. (TDF/FTC/EFV). Written informed consent was obtained in the participant’s chosen language, and clinical records reviewed to confirm eligibility. All participants who had a confirmed episode of VB while on the ADD-ART parent study were included in this sub-study.

### Study procedures

On study, participants were followed up every month for 12 months. Blood samples for VL and TFV-DP concentrations in DBS were obtained monthly. Demographic data were collected and questionnaires on self-reported adherence, HIV stigma [24, 25] and disclosure, screening for depression, anxiety, and substance abuse [26] were administered at baseline and 12 months.

At their baseline visit, a Wisepill^®^ Electronic Monitoring Device was issued to each participant. This provided continuous, EM over the study period. The device holds a 30-day supply of tablets, and a signal is sent to a secure website using cellular networks every time it is opened [12]. This was used as a proxy measure of adherence, operationalized as the percentage of study days for which an opening signal was received. To control for possible confounding, battery data voltage was accounted for, so days missing data due to a dead battery were not taken as a missed dose. Automatically generated text messages were sent to participants when the battery was low. Participants were contacted by study staff if the low voltage signal persisted for more than 7 days.

At each study visit, DBS were obtained using whole blood collected by venipuncture. Twenty-five microlitres of whole blood was pipetted five times onto a Whatman 903 ProteinSaver card and air dried. Cards were individually packaged with a humidity indicator and frozen at – 80 °C for batch shipment to the Colorado Antiviral Pharmacology Laboratory, where samples were processed for quantification of TFV-DP concentrations using a validated assay [6].

In conjunction with DBS specimens, whole blood was collected for Polymerase Chain Reaction (PCR) HIV-1 ribonucleic acid (RNA) VLs. Samples were assayed with the Roche COBAS AmpliPrep/COBAS TaqMan HIV-1 Test at the National Health Laboratory Service in Cape Town. For samples with a VL above 400 copies/mL, a repeat assay on the same specimen was run to confirm the result. Confirmed VB was defined as two results > 400 copies/mL from the same blood sample [6].

**Fig.1:**
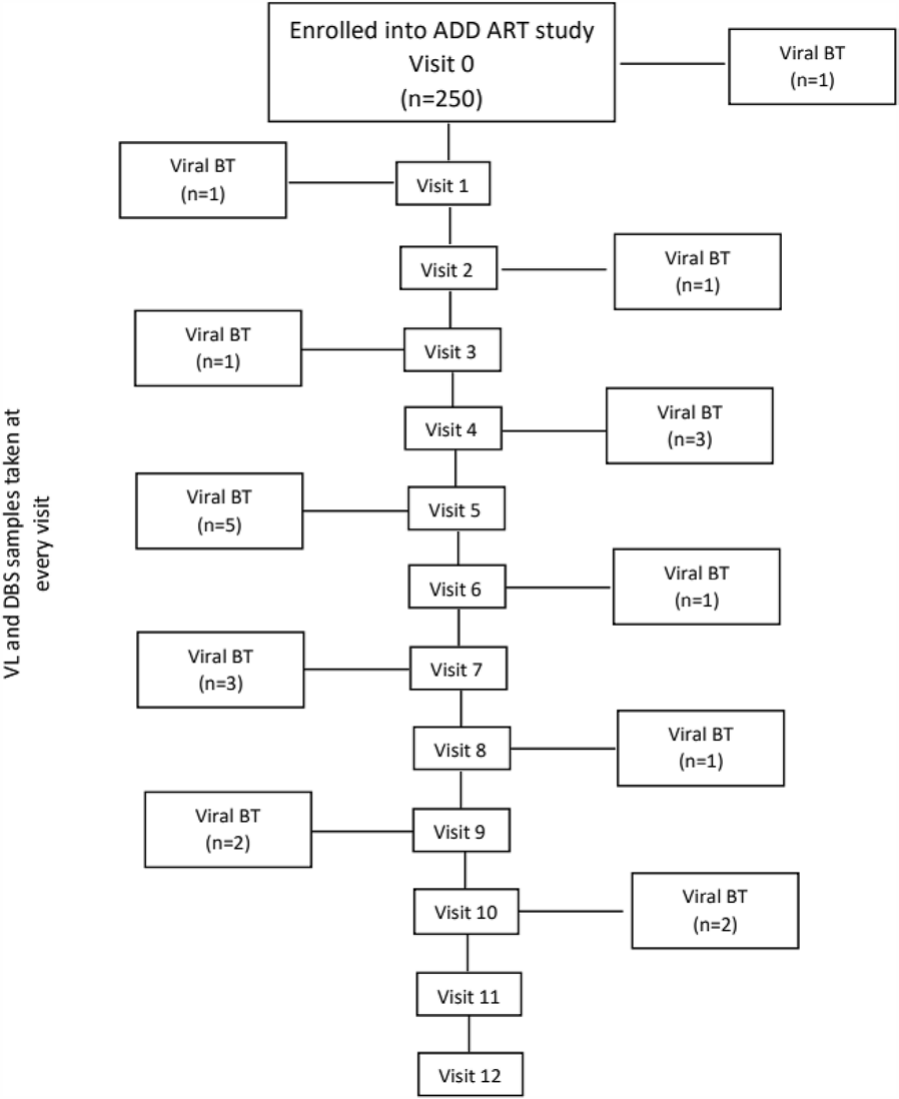
Diagram of enrolled participants on the ADD ART study and participants with confirmed viral breakthrough

Participants with confirmed VB provided whole blood samples for HIV-1 resistance testing at their next study visit (approximately 30 days later) Figure 1 below illustrates the visits at which participants experienced VB during the 12 month follow up (Fig. 1).

### Statistical analysis

Data analysis was performed using Stata Version 15. Summary statistics were generated for the full sample and for those with VB. Participants were censored after their first episode of confirmed VB. Medians and interquartile ranges [IQR] were reported for the variables of interest: 30 day EA prior to first VB and TFV-DP drug concentrations at VB. Time in months to first VB and HIV-1 VL at the time of genotyping were also reported.

We compared outcomes of HIV-1 genotype resistance testing by amplification and sequencing. Individuals were categorised as: 1. Did not amplify; 2. Amplified with no HIV-1 drug resistance mutations in evidence; 3. Amplified with HIV-1 drug resistance mutations; and 4. Genotype missed.

We used the ‘Did not amplify’ category as the reference group to compare median [IQR] TFV-DP by genotype outcome, using the Wilcoxon rank sum test.

For each category, TFV-DP and 30-day Wisepill^®^ adherence plots were computed with R statistical software (R Core Team, http://www.cran.r-project.org) using ggplot2, ggallin and dplyr packages. Plots are right justified, with data points beginning from the point of viral breakthrough baseline visits for each participant.

## Results

Of the 250 participants (195 of whom were female, 78% of the participants) from the original study cohort, 21 (8%) experienced VB at a median of 5 [4;7] months from study start and were included in this analysis. Most of the participants with VB were female (n = 18, 86%), with a median age of 33 [27;41] years. Baseline characteristics for the overall cohort are summarized in Table 1. Apart from employment status, there were no significant demographic differences between those who had VB and in those who remained suppressed in the 12 months of follow up.

**Table 1:** Demographic characteristics of the full and viral breakthrough cohorts

Variable	Full cohort	Viral breakthrough cohort	P value
n	250	21	–
Age (years): median (IQR)	34 (27;42)	33 (27;41)	0.27
Gender (female): n (%)	195 (78%)	18 (86%)	0.19
Level of education: n (%)			
Grade 1–7	20 (8%)	0	0.39
Grade 8–11	143 (57%)	16 (76%)	
Grade 12	75 (30%)	5 (24%)	
Grade 13+	11 (4%)	0	
Refused	01 (< 1%)	0	
Currently employed: n (%)	78 (31%)	3 (14.29%)	0.04
Food insecurity (any): n (%)	65 (26%)	7 (33%)	0.79
Disclosure: % people live with who know HIV status: median [IQR]	63(29;100)	59(25;100)	0.33
Stigma score: Scores from 0–12: median (IQR)	03 (1;5)	03 (0;5)	0.43
SAMISS score			
Any mental health diagnosis (anxiety/depression)	107 (43%)	8 (38%)	0.33
Any drug or alcohol abuse	126 (50%)	9 (43%)	0.24
Viral load at breakthrough log10 copies/mL: median (IQR)	–	4.0 (3.2; 4.5)	–

Resistance testing was completed for 18 of the 21 individuals. Data was missing for 3 cases (one death, one lost to follow up and one genotyping was missed in error.)

### Amplified with resistance (n = 5)

Amplification of virus with HIV-1 drug resistance mutations was identified in five of these individuals. (27.8%) Notably, only NNRTI-associated mutations were identified, namely: K103N, E138K and Y118H. (Table 2) Only the K103N mutation, which confers high level resistance to EFV, was identified in more than one case.

**Table 2:** Description of mutations identified at genotyping

Non-nucleoside reverse transcriptase inhibitor mutation	n
E138K	1
Y188H	1
K103N	3

**Fig.2:**
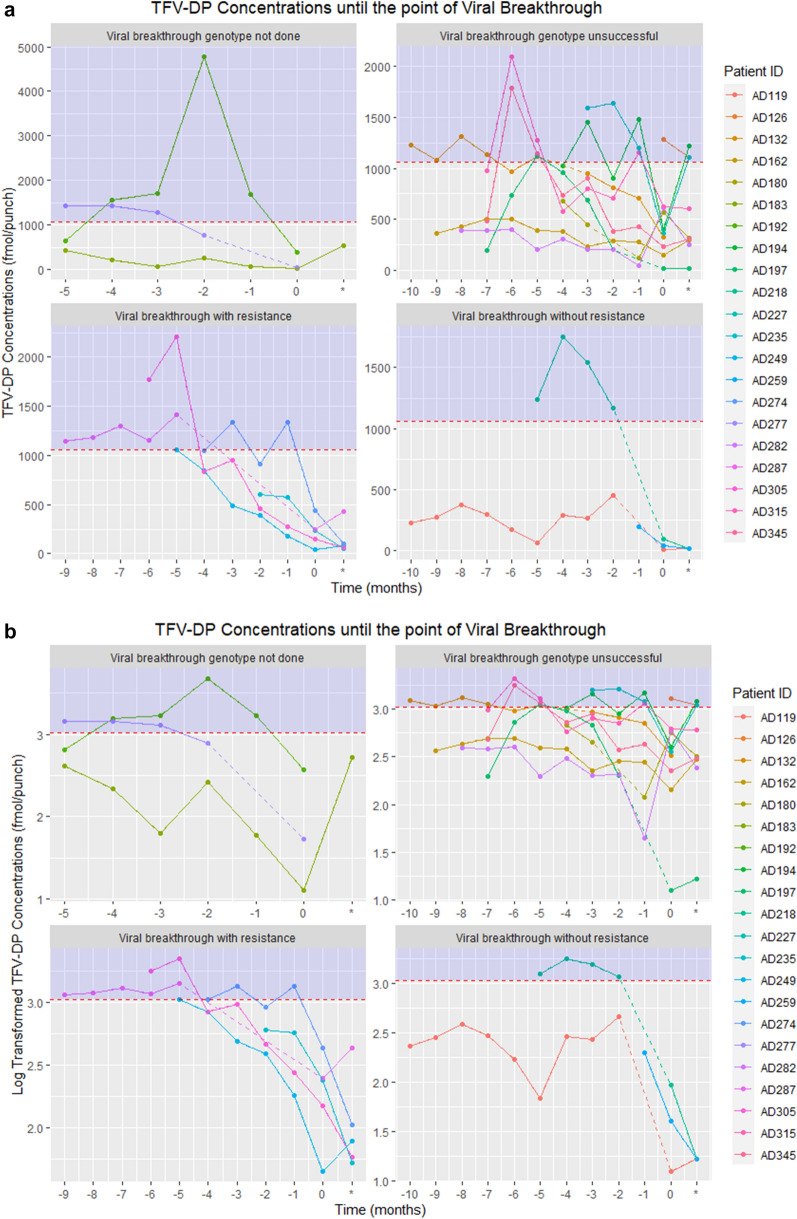
**a** Concentration of TFV-DP in DBS in participants with viral breakthrough according to resistance testing status: Viral breakthrough with resistance, viral breakthrough without resistance, viral breakthrough where genotyping was unsuccessful and viral breakthrough where genotyping was not performed. The graph is right justified, with time to first confirmed viral breakthrough in months, with month 0 at which viral breakthrough occurred. The red dashed line represents the median TFV-DP concentration in those with viral suppression. **b** Log transformed concentration of TFV-DP in DBS in participants with viral breakthrough according to resistance testing status

**Fig. 3:**
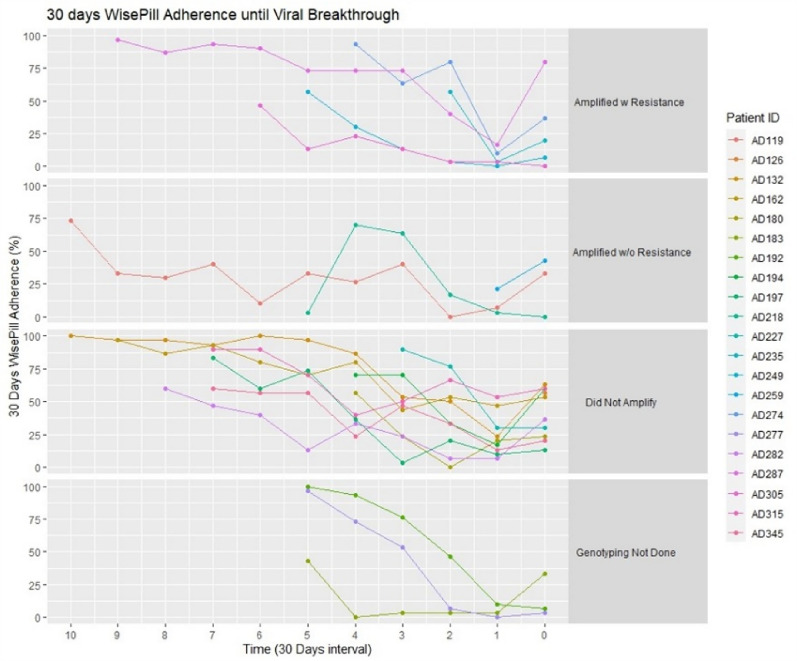
30 day Wisepill^®^ adherence in participants with viral breakthrough, according to resistance testing status: Viral breakthrough with resistance, viral breakthrough without resistance, viral breakthrough where genotyping was unsuccessful and viral breakthrough where genotyping was not performed. The graph is right justified, with time to first confirmed viral breakthrough in months, with month 0 at which viral breakthrough occurred

TFV-DP concentrations at breakthrough in this group were mid-range: of 241 [150;247] fmol/punch. However, given the predictive threshold TFV-DP concentration of < 400 fmol/punch [6], these concentrations may be considered as “low”. Thirty-day EA at breakthrough was 20 [7;37] %, the lowest across all categories of genotyped individuals (Table 3). TFV-DP concentrations and EA both trended down towards VB in those with amplified mutations (Fig. 2a, b). A considerable increase in EA occurred in 4 out of the 5 individuals between breakthrough and genotyping (Fig. 3). Four out of the 5 had TFV-DP concentrations < 400 fmol/punch [6] The fifth individual had a TFV-DP concentration slightly above this threshold, at 435 fmol/punch at their breakthrough visit (Fig. 2a, b).

**Table 3:** Description of genotyping results in participants with viral breakthrough (VB)

Genotyping result	n	Median [IQR] VL at genotype in log copies/ml	Median [IQR] time to VB in months	Median [IQR] TFV-DP at VB	Median [IQR] 30 day EM adherence at VB in %	P value
Did not amplify	10	2.0 [1.6;2.7]	7 [4;8]	380 [227;611]	45 [25;59]	0.035
Amplified with no HIV-1 drug resistance mutations in evidence	3	4.3 [3.9;4.7]	5 [1;10]	40 [17;94]	33 [17;38]	
Amplified with HIV-1 drug resistance mutations	5	3.2 [2.6;3.8]	5 [4;6]	241 [150;247]	20 [7;37]	
Genotype missed	3	–	5	54 [33;214]	7 [5;20]	
Totals	21	–	5 [4;7]	241 [53;401]	33 [13;53]

### Amplified with no HIV-1 drug resistance (n = 3)

Three of the 18 genotypes (16.7%) had no HIV-1 drug resistance mutations. In this category, median VL at genotype was the highest at 4 [4;5] log copies/mL, and median TFV-DP concentrations at VB were the lowest of all categories, at 40 [17;94] fmol/punch, (Table 3) with TFV-DP concentrations trending down towards VB (Fig. 2a, b). In 2 of these 3 individuals, data points for the TFV-DP concentrations from 2 months prior to VB were missing, so the individual trajectories for this category could not be fully characterized. The corresponding median EA for this category was 33% [17;38] (Table 3).

### No amplification at genotyping (n = 10)

The remaining 10 individuals (55.6%) did not have viral amplification at the genotyping visit due to re-suppression of the VL, as depicted in Table 3.

Median TFV-DP concentrations at breakthrough were the highest: 380 [227–661] fmol/punch (p = 0,035) in the re-suppressed group. The corresponding EA median was 45% [25;59], also the highest EA across all categories (Table 3).

## Discussion

This study describes the TFV-DP concentrations in DBS and EA at the time of VB in a South African cohort receiving a TDF-based ART regimen. All enrolled individuals were on a single tablet coformulation containing TDF/FTC/EFV. A total of 21 out of 250 participants (8.4%) experienced VB (defined as a confirmed VL > 400 copies/mL) during the 12 month study period. The range for TFV-DP concentrations associated with sustained viral suppression have previously been reported, and a threshold TFV-DP value of 400 fmol/punch was found to be predictive of future viral breakthrough in this cohort [6]. This association was stronger with TFV-DP concentrations measured 2 months before VB [6].

Among the individuals with evidence of NNRTI-associated resistant mutations (n = 5) the TFV-DP concentrations trended downwards towards the visit at which VB occurred which is consistent with a decrease in adherence that precedes the development of resistance. Corresponding EA at breakthrough was also low, but not completely absent in these participants, which was consistent with the TFV-DP concentrations in DBS. In comparison, individuals (n = 3) whose HIV-1 amplified without any resistance mutations (wild type virus) had lower concentrations of TFV-DP than those with mutations, which supports the premise that some degree of drug exposure is required to promote drug resistance.

Our findings are similar to those from another South African cohort on a NNRTI based, TDF containing regimen. In that study, Castillo-Mancilla et al. demonstrated that individuals with evidence of HIV-1 drug resistance had higher TFV-DP concentrations compared to those without evidence of resistance on genotyping [21]. However, in this study, the study endpoint was virologic failure with a VL > 1000 copies/mL, and adherence was only quantified using medication possession ratio. Congruent with our study findings, a stepwise decrease in TFV-DP was identified in VB with and without resistance. Individuals with virologic failure and evidence of drug resistance had higher median TFV-DP concentrations than those without resistance, when compared to those with a genotype that did not amplify. Median TFV-DP concentrations (almost 90% less than those who did not amplify aligned with relative non-adherence in those who amplified without drug resistance[21]. In comparison, our research utilized EA with TFV-DP concentrations to illustrate adherence across categories. Unlike the findings from Castillo-Mancilla and colleagues, median EA in the group without drug resistance was found to be higher than in the resistance category, whereas TFV-DP was lower in the group without resistance. Contrasting with our findings, a study conducted in a similar population of PWH in the US found higher TFV-DP concentrations in its 10 participants with HIV-1 resistant mutations. (TFV-DP median concentration of 956 [407–1510] fmol/punch.), but still lower than the concentrations associated with the highest odds of viral suppression [1, 27]. HIV-1 mutagenesis in regimens with a higher genetic barrier to resistance may be slower [28] drugs with a high genetic barrier to resistance (like DTG) bind more tightly to HIV and can keep working even after he virus has undergone mutagenesis.

This mechanism may explain our findings of unsuccessful viral re-suppression, and consequent drug resistance after improved adherence. EM and TFV-DP in DBS may be more useful in individuals on these regimens [20].

A strength of our research is the utilization of two objective metrics of adherence for a more comprehensive illustration of trajectories in those who had viral resistance. Given that adherence was monitored in real-time, we could illustrate longitudinal courses up until the point of breakthrough, despite the missing data on the TFV-DP time course. Building on the findings of a predictive threshold of TFV-DP for detecting VB [6], this research iterates the usefulness of routine drug concentration measurements in the clinical setting. A single annual VL measurement is essentially a retrospective glance at an individual’s adherence. Relying solely on this tool means missed opportunity to intervene prior to the development of virologic failure and possibly, drug resistance.

We also acknowledge several limitations to our research. First, our study sample was small, and three of the 21 individuals had not undergone resistance testing. Furthermore, multiple missing data points represented in our TFV-DP time course limit the characterization of individual trajectories within each of the genetic testing outcome groups. However, these missing data points on the TFV-DP plots were due to missed study visits when DBS were not obtained. The small cohort with VB may be partly attributed to the Hawthorne effect- modification of adherence behaviour in response to being observed during the study, although this effect is unlikely to have lasted for the duration of the study [29]. ADD-ART was an observational study without any formal adherence support intervention. Participants attended monthly visits and were seen by clinical staff. Participants were only texted if the device had low or no battery signal. Although this likely contributed to better adherence in the overall cohort, 21 individuals with breakthrough were indeed identified. An unexpected finding was the identification of only NNRTI associated viral mutations. Existing literature describes the effect of reverse transcriptase inhibitors and accelerated HIV-1 mutation rates [30, 31]. A follow up study examining the cases of successive episodes of virologic failure may shed light on the evolution of drug resistance in individuals.

## Conclusion

This study illustrated a step-wise inverse relationship with TFV-DP concentrations and VB with drug resistance in PWH on a NNRTI based regimen in South Africa. Two objective measures of ART adherence, namely, TFV-DP concentrations and EA, showed low (but not completely absent) ART adherence in those who developed drug resistance. Our findings add to the evidence of the utility offered by these measurements in predicting future VB, and drug resistance. In routine clinical care, monitoring TFV-DP trends could give clinicians the opportunity to safety-net individuals meeting the threshold for low TFV-DP concentrations (less than 400 fmol/punch) [6] for timely adherence support to prevent resistance [23]. The identification of only NNRTI-associated mutations-two major primary mutations (K103N and Y188H) and one minor drug mutation (E138K) justifies expediting the transition to dolutegravir containing regimens to promote treatment longevity. To date, quantification of TFV-DP in DBS still requires technical expertise. However, further evaluation into the feasibility of its clinical use, even as a proxy for HIV-1 resistance testing, could extend its application from research to a clinical setting.

## Data Availability

Data are available from the authors upon reasonable request.

## Abbreviations

ART: Antiretroviral therapy
TFV-DP: Tenofovir diphosphate
DBS: Dried blood spots
PWH: Persons with HIV
TDF: Tenofovir Disoproxil Fumarate
VL: Viral load
VB: Viral breakthrough
HIV-1: Human immunodeficiency virus type-1
NNRTIs: Non-nucleoside reverse transcriptase inhibitors
EM: Electronic monitoring
EA: Electronic adherence
PCR: Polymerase chain reaction
RNA: Ribonucleic acid
IQR: Interquartile ranges
